# “It’s complicated” - talking about gout medicines in primary care consultations: a qualitative study

**DOI:** 10.1186/s12875-016-0515-y

**Published:** 2016-08-19

**Authors:** Caroline Morris, Lindsay Macdonald, Maria Stubbe, Anthony Dowell

**Affiliations:** Department Primary Health Care & General Practice, University of Otago, Wellington, 23a Mein Street, Wellington, New Zealand

**Keywords:** Communication, Consultation, Gout, Medicine, Primary care

## Abstract

**Background:**

Gout is the most common form of inflammatory arthritis. It is associated with substantial co-morbidity and often managed in primary care. A greater understanding of the communication process between patients and healthcare professionals provides one way of improving the management of this condition. This paper describes communication about gout medicines and treatment between patients and primary care health professionals during routine consultations.

**Methods:**

Video-recordings of 31 individual healthcare consultations between patients and a range of primary care practitioners (general practitioners, practice nurses, podiatrists, dietitians) from an archived database were reviewed. Consultations that encompassed any discussion about gout medicines and treatment were included (*n* = 27) and were not solely restricted to those where gout was the presenting complaint. Themes were derived from an inductive qualitative analysis, from clinical and linguistic perspectives, based on the conversation between patients and practitioners about medicines and visual observation of these interactions.

**Results:**

A number of factors were identified that had the potential to impact on the optimal management of gout in primary care. These included level of patient knowledge, patient attitudes to medicines, and the attributes of practitioner communication with patients. The latter related to the style of delivery and content of the information provided, and the ability of practitioners to make use of opportunities that arose to discuss these issues.

**Conclusions:**

Patients with gout communicate at varying levels of complexity with a diverse range of primary care healthcare professionals about the treatment of their condition. It is important that all practitioners engaging with gout patients in this setting are knowledgeable about the current management of gout, provide clear, consistent and accurate messages, remain aware that these messages may need repeating over time, and are supportive of patients’ medicine-taking preferences.

## Background

Gout is recognised as the most common form of inflammatory arthritis and is often associated with substantial co-morbidity [1]. This condition, usually managed in primary care, is closely linked with diabetes and chronic kidney disease and is an independent risk factor for cardiovascular mortality [2]. The clinical and economic burden of the disease is substantial [3, 4].

Some aspects of gout management appear straightforward and there is a plethora of management reviews and treatment guidelines in existence globally [1, 5–9]. Non-steroidal anti-inflammatory drugs (NSAIDs) are one of the main medicine groups used for the management of acute flares, with colchicine and steroids providing alternative treatment options. A target serum uric acid level of less than 0.36 mmol/L is aimed for and allopurinol and other urate-lowering therapies are indicated for the chronic management of gout.

The burden of the disease is rising, [3] and aspects of management remain challenging. Differences in primary care management strategies have been widely noted between routine practice and both reported management and recommended guidelines [10–15].

Differing patient and physician perceptions of gout have been identified as one of the challenges to management in primary care [16]. Issues identified include patients experiencing difficulty in obtaining information they perceive as directly relevant to them, [17] self-diagnosis and treatment by patients, [18] a lack of patient knowledge about gout medicines, [19–22] a lack of practitioner knowledge about treatment guidelines and recommendations [21] and a reluctance on the part of some practitioners to offer preventive medicines as a long-term solution, with some making an assumption that patients would prefer to be treated for an acute flare rather than take long-term medication [21].

While some previous studies have attempted to address views about gout treatment from both patient and practitioner perspectives, [21, 23] there is no research that investigates directly observed conversations in primary care consultations. Existing findings therefore rely on people’s abstract perceptions or retrospective reports of healthcare encounters. We describe communication between patients and primary care practitioners about gout medicines and treatment in the context of routine consultations. We identify how patient and practitioner perceptions are brought into play in the course of natural interactions between them. The prevalence of gout, due to its association with cardio-vascular disease and diabetes, is likely to increase further. A greater understanding of the communication process will help determine how the management of this condition can be improved.

## Methods

### Data source

Data were derived from the Applied Research on Communication in Health (ARCH) Corpus at the University of Otago, Wellington, New Zealand [24]. This comprises a searchable digital collection of healthcare interactions and related data for use in interactional and narrative analysis. The data in the Corpus has been collected in the course of a series of funded projects since 2003. These studies include the ‘Interaction Study’ (IS), a study exploring clinical decision-making when rationing is explicit; the Tracking Study (TS), a study exploring communication processes throughout a single complete episode of care of patients referred from primary to secondary care; the Diabetes Study (DS), a longitudinal study tracking the contact of newly diagnosed patients with Type 2 diabetes with healthcare professionals over a six-month period. Data are permanently archived for use by authorised researchers.

As the data contained in the Corpus has been collected on a project-by-project basis it does not claim to be ‘representative’ in the statistical sense. It is, however, representative in the sense that the video-recordings were made in the course of ‘practice as usual’. They are therefore typical of routine interactions occurring in the New Zealand healthcare setting.

### Logging of data in the ARCH Corpus

The digital video-recording for each consultation is logged by a trained research nurse onto a template summarising key clinical content (e.g. presenting problem, other clinical issues discussed, treatment with medicines) and a time-coded sequence of key events and topics discussed. Each consultation is subsequently transcribed in full using linguistic transcription conventions to capture both the words spoken and selected non-verbal cues (e.g. typing of notes on the computer) to assist in interpretation of the transcribed speech. The Corpus has a custom-designed information management system.

### Search strategy for identifying gout consultations

When sampled, the Corpus included 418 video-recorded interactions between patients and practitioners recorded between 2004 and 2011. Consultations occurring in primary care and consultations where patients had given consent for secondary analysis of their data were included, resulting in a subset of 337 eligible consultations. These comprised 247 individual patients, 30 general practitioners (GPs), 31 nurses and 15 other practitioners.

A query was then run on the information held within the database using the keyword “gout”. A second search incorporating a list of key gout-related medicines was run, but did not yield any additional consultations.

### Consultations included in the analysis

The search identified 31 consultations, 27 of which were included in the final analysis.

The majority of consultations identified came from those archived as part of the DS (*n* = 20). Gout was not, therefore, the primary focus of these encounters; many gout-related conversations stemmed from enquiries made by practitioners that resulted directly from application of the nationally agreed protocol for diabetes care. Despite this, discussion about gout medicines of varying length and complexity was a feature in the majority of these consultations (*n* = 16). In the remaining four, there was no mention of medicines in the context of gout; these consultations were therefore excluded from further analysis. As this study tracked patients over a six-month time-frame, the same patient was sometimes involved in more than one consultation in the sample and potentially more likely to discuss gout medicines with a practitioner if they had talked about them in a healthcare encounter in the recent past.

Contextual information about the 11 GP consultations derived from the IS study (*n* = 5) and the TS study (*n* = 6) is provided in Table 1.

**Table 1:** GP consultations that were not part of the DS (*n* = 11)

Description	Number
Gout was the patient’s presenting complaint	2
Gout was the presenting complaint in the patient’s previous consultation (not part of the Corpus archive) and was followed up by the GP on this occasion	2
Gout discussed in the context of a medicine that had been previously prescribed for this condition	7

*GP* general practitioner

### Patient and practitioner demographic characteristics

The final dataset of 27 consultations used for analysis encompassed 17 individual patients (male *n* = 12, female *n* = 5; age-range 36 to 67 years) consulting with 15 different practitioners. The number of consultations per patient ranged from one to eight, with the majority of patients (14/17) having only one included consultation. Patients interacted with a diverse range of professionals (GPs *n* = 9, nurses *n* = 3, podiatrists *n* = 2, dietitian *n* = 1).

### Data analysis

This was an exploratory descriptive study based on direct observation of gout-related interactions from both clinical and linguistic viewpoints. Our aim was to identify and report on the range of patient-practitioner models of engagement around medicines use as these emerged from the data, with no preconceived assumptions.

Themes identified from the overall dataset were derived via an iterative process of inductive qualitative analysis [25] of the medicine-related gout conversations between patients and practitioners, with visual observation of these interactions complementing the textual analysis of the transcripts. CM identified and coded all mentions or sequences of talk relating to the use of medicines in the management of gout in the transcripts. Video-recordings of consultations were then viewed to confirm and enrich the content analysis of the transcripts. Initial coding included the location within the consultation, who (patient or practitioner) initiated the conversation, the types and names of medicines discussed, the context in which that discussion took place and any significant non-verbal interactions. The video-recordings and transcripts were then re-reviewed to identify any other medicine-related conversation to help provide additional insights and context for the gout-related medicines communication. CM and AD reviewed and discussed the initial themes derived from data analysis and interpretation of data from their clinical perspectives (pharmacy and general practice), with disagreements resolved by consensus. LM and MS subsequently reviewed the data and themes derived from a linguistic and interactional perspective. All authors reviewed and agreed the final themes.

## Results

### Communication about gout medicines

Interactions about gout treatment occurred at various levels of complexity, taking many different forms. Some focused solely on the treatment of acute flares with NSAIDs, colchicine or steroid injections, some solely on preventive treatment with allopurinol, while others encompassed both acute and chronic management.

Three overarching themes emerged from our data. These themes (and sub-themes where applicable) are shown in Table 2 and described more fully below. Abbreviations used in the quotes are as follows: GP = general practitioner; NS = nurse; PT = patient; POD = podiatrist.

**Table 2:** Overarching themes and sub-themes identified from the data

• Level of patient knowledge • Patient attitudes to medicines • Attributes of practitioner communication - Delivery and content of information provided - Taking the opportunities presented	

#### Level of patient knowledge

We identified a number of instances where patients displayed a lack of important knowledge about medicines used for gout. NSAIDs, a medicine class with significant potential to cause harm if inappropriately used, caused confusion for a number of patients. In this example a patient with a long-standing diagnosis of gout had been unsuccessfully self-treating an acute flare with an insufficient dose of a medicine purchased over-the-counter:

GP: so, you’d been taking lots of … those ‘mini’ Voltarens™ [diclofenac 25 mg strength], and so we switched you on to the one hundreds [100 mg strength] ... and when you started on those initially you said there was a bit of improvement …” (IS-GP02-14)

The GP then listens carefully and attentively to the patient as they describe the beneficial effect that this increase in dose has had on their symptoms over time.

In addition, some patients showed a lack of understanding of generic and brand (™) names, not realising that the same medicine could have two different names. In this consultation, a patient with a history of hip pain managed with an NSAID is newly diagnosed with gout. The GP spends considerable time explaining the causes and symptoms of gout before mentioning treatment with medicines:

GP: “… I’ll give you some diclofenac, … it’s good for your gout and also for the other pain

PT: and can I have some Voltaren™ [diclofenac] please?

GP: It’s the same thing as Voltaren™ [diclofenac]

PT: Is it?” (DS-GP29-01)

The following patient with a confirmed diagnosis of gout is already willingly taking a low dose of allopurinol as preventive therapy. However, this is not currently adequately managing their symptoms. The GP explains the reason why they are unable at this point to increase the dose:

Joint consultation with nurse and GP:

GP: “… now you know those allopurinol tablets for the gout, you still taking them?

PT: yep, cos I wanted to prevent the gout

GP: the problem [is] that’s quite a low dose and we probably need to increase it, but I can’t increase it when you’ve got acute gout because it gets worse so … what we’ll do is leave you on that dose and increase it in a couple of weeks when it’s better.” (DS-NS13-01c_GP18)

The patient smiles indicating that they are comfortable with this approach and understanding of the reasoning behind it. The following excerpts from subsequent consultations with this patient and other practitioners appear to show that a patient’s understanding may evolve over time and that confusion may creep in as a result.

Consultation with dietitian two weeks later:

PT: “They haven’t put me on [more] of the medication [allopurinol] because this can enhance more gout, so they just said to me to stick on what I’ve got and hopefully when I get rid of the gout they’ll increase it, so it can lower the [number of] times I have gout.” (DS-HP06-01b)

The patient had been given information by one practitioner (GP) and then recited that same information back to another practitioner (dietitian) a short time later. Two weeks after this, the patient recalls that they were to stay on the same dose, and reconsiders their situation in light of their worsening symptoms. While doing so they are also now questioning the possibility that allopurinol can exacerbate gout.

Consultation with podiatrist one month after the GP consultation:

PT: “It [gout] started from my toes round to my ankle and then it just stayed there for a while and it just slowly headed up … [and] they told me to keep on it [allopurinol] … so I stayed on that and I thought that was triggering it because that’s the longest I’ve ever had gout … [and] I wasn’t quite sure … and I was questioning myself ‘Should I stop it?’ cos I don’t know if that was triggering it worse

POD: but it’s a treatment for gout so you wouldn’t think it would actually exacerbate your symptoms or pain …” (DS-HP07-01b)

This consultation series also shows that in primary care teams with multiple healthcare professionals, advice about medicines may be given by professionals other than the prescriber. In this instance it would have been helpful if the podiatrist could have appropriately addressed the patient’s understanding. However, in the final quotation, the podiatrist seems unaware of the action of allopurinol and inadvertently creates a further source of confusion potentially undermining the patient’s knowledge and understanding of gout treatment.

#### Patient attitudes to medicines

It was clear that how patients perceived medicines sometimes impacted on the way in which their gout was treated. One patient with previously diagnosed gout identified “I don’t like tablets” as a general statement at the beginning of a consultation and then segued straight into talking specifically about gout:

PT: “… and I said before, I told you with my gout I don’t want to take any more tablets …

GP: When did you last have a flare of gout?

PT: Well, sometimes I feel it is a little bit sore on my elbow but I don’t take anything - it comes and goes …

GP: “… your uric acid level in your blood, that’s the one that’s associated with gout is still high, so I think if you do start having regular flare-ups then we should look again at putting you back on allopurinol to bring that uric acid level down.” (DS-GP19-02b)

While it was unclear what the patient’s uric acid level was or whether allopurinol therapy at this point was justified, clinically, the impact of the condition on the patient’s life appeared manageable without the use of medicines. It is possible that the GP may have judged the level of clinical severity against the patient’s desire to be tablet-free and they appeared accepting of the patient’s preference at this point in time. While there was no discussion about the reasons for the patient’s reticence to take medicines, the possible future need for treatment with tablets was brought clearly into the open by the GP, with tacit approval from the patient indicated by a nodding of their head and a verbal acknowledgment in the form of “yeah, yeah”.

Another patient was fully aware of the potential adverse impact that taking an NSAID for her long-term gout symptoms could have on a co-morbid condition. Although her specific circumstances dictated that she took the medicine anyway, she had developed a personal strategy in an attempt to minimise the risk:

NS: “What are you taking in terms of pain relief?

PT: Well, I actually ran out of what [the GP] gave me … and all I had was diclofenac … so what I was trying to do was half it [the dose] because I was in so much pain because I know that the diclofenac triggers my asthma” (DS-NS13-01d_GP18)

There is no discussion or negotiation about alternative options that may be more suitable for the patient, nor is the patient able to glean what the nurse may think by either verbal or non-verbal means. The consultation moves swiftly on to codeine, another medicine that the patient is taking for pain relief, and its tendency to cause constipation:

PT: “so then I had, what do you call it, codeine

NS: codeine doesn’t help the constipation either” (DS-NS13-01d_GP18)

### Attributes of practitioner communication

#### Delivery and content of information provided

Communication style varied across the consultations. Many practitioners asked open questions, clearly listened to the patient’s view, provided clear, accurate information, and confirmed that the patient understood this information and was comfortable with the final decision. In this example the patient presented to the GP because they had run out medicines. This prompts a general discussion about their medicines leading to the patient describing symptoms they knew to be gout:

GP: “… getting a good dose of diclofenac would be probably the most useful thing; so this is your first gout attack for a long, long time isn’t it? So I don’t think we need to look at the allopurinol thing

PT: well, no cos I mean this attack, I totally knew [the cause], it just didn’t occur to me at the time, it was just silly, but yeah this [attack] isn’t a mystery” (TS-GP09-05)

There was clear agreement between them, with the GP smiling and both parties verbally acknowledging that they were happy with this approach by saying “mm mm”.

In another consultation a substantial amount of discussion, initially raised by the GP, took place around the use of colchicine in a patient with gout that had previously proven difficult to manage. Effective clear communication with the patient occurred resulting in a clear management plan agreed by both parties. The only contentious issue here might be the use of the word “prophylaxis”; something that may not necessarily be understood by the patient:

GP: “… allopurinol is probably an option, but [the specialist] thought you should stay on colchicine … which is what you’ve done isn’t it? One tablet twice a day for the prophylaxis and I think if that’s doing the trick and not giving you symptoms then that’s fine … if that turned [out] for any reason not to be suiting you in terms of prevention; either not effective enough or giving you side effects, then there are the other things that we can try

PT: I’m actually only taking one a day, it seems to be effective so then I guess I could

GP: if one a day is doing it that’s fine [but] you can easily up it to two if you’re getting any breakthrough - it would be fine.” (IS-GP02-03)

The patient tacitly indicates by nodding his head that he agrees with the GP.

All of the podiatrist consultations (*n* = 4) were with patients with diabetes and featured practitioner engagement with the patient around medicines to some degree. These consultations were relatively lengthy (between 20 and 30 min) compared to GP consultations and talk about medicines was usually used as a time-filler following what seemed to be uncomfortably long gaps of silence for the practitioner. The following extract took place 10 min into a consultation where the podiatrist had been asking questions or providing information about diabetes with limited response from the patient:

POD: “… [gout] that’s a type of arthritis [that] tends to run in families … it can be quite painful gout are you on medication for it?

PT: Yes” (DS-HP10-02a)

#### Taking the opportunities presented

While a small number of ‘lost’ opportunities to discuss gout and gout medicines were identified across the dataset, there were many excellent examples of practitioners making good use of opportunities that presented themselves by responding to ‘openings’ provided by patients.

In this example the patient raises the issue of gout, identifying that he had been given an unnamed “injectable treatment” abroad in the past. Although the patient clearly articulates that gout is not currently a problem, the GP seamlessly picks up on the opportunity to explain the rationale for his current treatment approach offering reassurance that alternative options exist should the patient’s circumstances change:

PT: “…I do get gout … but it normally goes away by itself. I think the last time would have been last year sometime …

GP: I feel we’ve got more than enough information right now to know what the next step should be and at this stage, because things are not that bad, I’m saying I’d quite like to be very conservative in this and not push you know lots and lots of treatment, but we’ve got it there waiting if things got a bit [clinically] worse or didn’t improve as far as these [uric acid] numbers are concerned” (DS-GP01-04)

The patient listens carefully to the explanation, smiles, maintains eye contact and nods their head in acceptance.

Another example shows the potential benefit of coordinated multidisciplinary care. This patient had expressed concern about their gout pain to the nurse in an appointment the previous week. The nurse then reintroduces this issue in their second consultation, discussing the patient’s symptoms with them and identifying that there was a need to involve the GP further in their gout-related care. In this instance, having been provided with information about a new treatment option for their symptoms the patient very clearly verbally articulates that this is something they would like to try.

NS: “I had a wee chat with [GP’s name] … you had a concern about your toes about the gout … and she says that if you’re having more than two episodes in a year

PT: oh yes definitely

NS: oh okay, she said then you probably need some treatment which you carry on taking long-term because it helps to prevent [gout]

PT: oh I would - it’s always at the back of my ankle and … it gets right here [pointing at foot] and I can’t walk on it and stuff like that, six times maybe seven times a year easily and they all swell up and the other place I get it is on my side of my toes …

NS: have you got those symptoms at the moment? No okay, alright so I probably need to leave a message for [the GP]” (DS-NS13-01b)

However, not all patient-practitioner gout interactions resulted in any significant discussion about the condition. Sometimes another potentially more pressing problem or patient concern becomes the focus of that part of the consultation and the moment and the potential opportunity has passed.

Only one example of an opportunity being truly missed was identified from the dataset. This practitioner phrased her question about a gout medicine in a closed way that did not invite debate or discussion as she simultaneously turns her back to the patient and prints out a computer generated blood test form:

NS: “your allopurinol, you know about it don’t you? It’s the same. So that’s a fasting blood test [form], first thing in the morning for fasting …” (DS-NS27-02)

There is silence while the form is printing and once this is done the nurse hands it to the patient, explains what fasting means, advises them when they should schedule their next visit and the consultation is closed.

## Discussion

Our study is the first to describe communication about medicines for gout treatment in the context of routine consultations in primary care. We identified three overarching themes from the data; level of patient knowledge about gout medicines, patients’ attitudes towards medicines, and the attributes of practitioner communication. Each of these inter-linking themes has the potential to impact either positively or adversely on the successful management of gout. Optimising the manner, and avenues, by which information is shared with patients has the potential to improve patient knowledge and understanding, and promote clinician-patient agreement. A link between good communication and positive health outcomes has been previously described in the literature [26].

The strength of our study is that it enabled us to see, hear and review the interaction between patients and their practitioner in real encounters, rather than relying on the abstract views or retrospective opinions of each party. As some of the included consultations formed part of a longitudinal study we were also able to review encounters with a range of practitioners and gain insight into how patients’ knowledge and attitudes may alter over time. Although data were drawn from an archived database rather than a study specifically related to gout, the use of a video-recorder may have introduced an artificial bias, with participants behaving differently from ‘normal’ due to its presence. However, although all parties appear cognisant of a camera’s presence early on in a consultation, this effect can be observed to wane as the business of the consultation progresses [27].

It was concerning that some patients lacked basic knowledge about the main medicines used for gout treatment. This has clear implications for the quality and safety of medicines use. In line with earlier work, [19, 20] this study revealed lack of knowledge related to the appropriate NSAID dosing for an acute gout flare and confusion around NSAID generic and brand names. Some patients also lacked a clear understanding of the specific and unusual issues around the initiation of allopurinol therapy; an area which has been previously identified as a source of patient confusion [19, 20, 28].

It was evident on following a single patient through a series of consultations that information may need repeated explanation to ensure that understanding remains consistent over time. Information that appeared to be unproblematic when initially given became less clearly understood by the patient as time progressed and other experiences intervened. All healthcare professionals that may interact with patients, including community pharmacists, have a potentially valuable role to play in facilitating patient understanding and hence the safe and effective use of medicines.

The level of knowledge about gout treatment may impinge on patients’ attitudes towards gout medicines and the likelihood of patient-practitioner concordance [29]. There were examples in the present study of patients taking medicines ‘differently’ to the way the practitioner expected and a reticence expressed, by some, to taking preventive medicines long-term. To be considered for preventive treatment patients will have been previously symptomatic, although the level of pain experienced by an individual may vary widely. It is therefore not surprising that here, and in previous studies, [19, 23] patients have admitted to failing to take medicines both unintentionally (they were unaware of how it should be taken or simply forgot) and intentionally (they had no desire to take a medicine every day) [19, 23]. It is notable that one patient continued to take a medicine to treat gout despite the fact that it exacerbated a co-morbid condition, identifying that patient values and judgments around medicine side-effects may differ from their practitioner’s.

Communicating in an open way and engaging the patient in a discussion about treatment options is likely to increase patient knowledge about the rationale behind their treatment and potentially promote agreement and adherence to a treatment plan that takes account of the patient’s beliefs and wishes [29]. Although some patients may ultimately choose to defer a treatment decision to their healthcare professional, the majority desire some level of involvement in the decision-making process [30]. Patients are only able to make an informed and evaluated decision on how they personally wish to engage with medicine taking or sign-up to a suggested treatment plan if they have appropriate and accurate information on which to base their decision. It is therefore of note that gout patients have previously identified that they did not always understand the information that GPs provided and have suggested that practitioners should explicitly confirm their understanding [19]. Furthermore, as in our study, potentially important mismatches in perceptions between patients and healthcare providers (including doctors, nurses, podiatrists) about the effectiveness and place of specific medicines in gout management and the adequacy of information provided have been shown to exist [23].

The present study reviewed consultations with a range of different primary care health professionals. It was notable that medicines were a feature of consultations across all professional groups, although, not surprisingly, medicines-related talk occurred to a greater extent with GPs who are the prescriber of medicines. It is, however, vital that all practitioners engaging with gout patients are knowledgeable about the up-to-date management of this condition and provide clear, consistent and accurate messages to avoid the potential for misleading or confusing patients.

While our findings potentially have relevance to other musculoskeletal conditions, it is possible that communication between patients and practitioners about gout treatment may differ from, for example, osteoarthritis [31]. In contrast to this condition, gout can be effectively managed using preventive therapy and is far less likely to be perceived as a normal part of getting old.

Our study was undertaken in the context of routine clinical practice. It thus allowed us to view the communication process within the real time constraints under which practitioners undertake their daily work. Furthermore, consultations were not solely for gout providing, for the first time, an indication of how this issue is routinely raised in primary care practice. Overall, there were many examples of good communication; practitioners often use open questions, are supportive of their patients’ medicines-related preferences and seize opportunities that present to discuss gout and its treatment.

The difficulty of managing patients with gout and its associated co-morbidities in a short consultation is a key challenge to the optimal management of gout in primary care [16]. Given that medicines are a key feature of gout management and that GP consultation time constraints are unlikely to be easily overcome, a multidisciplinary team approach to care via primary care gout clinics including pharmacist input has been recently reported [32].

## Conclusions

Patients with gout communicate at varying levels of complexity with a range of primary care practitioners. All practitioners who interact with gout patients need to be able to provide patients with clear, consistent and accurate messages about gout treatment as the need arises, be aware that these messages may need repeating over time, and be supportive of patients’ medicine-taking preferences for this condition.

## Abbreviations

ARCH: Applied Research on Communication in Health
DS: Diabetes study
GP: General practitioner
IS: Interaction study
NS: Nurse
POD: Podiatrist
PT: Patient
TS: Tracking study
